# *Hmox1* Deficiency Sensitizes Mice to Peroxynitrite Formation and Diabetic Glomerular Microvascular Injuries

**DOI:** 10.1155/2017/9603924

**Published:** 2017-11-22

**Authors:** Olivia Lenoir, François Gaillard, Hélène Lazareth, Blaise Robin, Pierre-Louis Tharaux

**Affiliations:** ^1^Paris Cardiovascular Centre (PARCC), Institut National de la Santé et de la Recherche Médicale (INSERM), Paris, France; ^2^Université Paris Descartes, Sorbonne Paris Cité, Paris, France; ^3^Nephrology Division, Georges Pompidou European Hospital, Assistance Publique-Hôpitaux de Paris, Paris, France

## Abstract

**Objective:**

Indirect evidence suggests a role for heme oxygenase-1 (HO-1) in limiting diabetic vasculopathy. The goal of this study was to assess the role of HO-1 in the development of microvascular lesions within glomeruli during diabetes mellitus using a mouse model with specific alteration of the *Hmox1* gene.

**Approach and Results:**

The effects of *Hmox1* haploinsufficiency were studied as a means of assessing the intrinsic contribution of HO-1 in the development of renal microvascular lesions during diabetes. Renal function and histology were analyzed 10 weeks after diabetes induction with streptozotocin. Diabetic *Hmox1*^+/−^ mice showed higher levels of albuminuria and blood urea compared to their wild-type diabetic littermates. More severe glomerular microvascular lesions were also observed in the diabetic *Hmox1*^+/−^ mice. This was associated with a renal increase in the expression of the oxidative stress marker, nitrotyrosine.

**Conclusions:**

Genetic *Hmox1* partial deficiency is sufficient to sensitize mice to the development of diabetic glomerular microvascular lesions. HO-1 exerts antioxidant effects in the kidney during diabetes mellitus. These have protective effects on the development of glomerular endothelial injury.

## 1. Background

Diabetic nephropathy (DN) is a major microvascular complication of diabetes [1]. Progressive loss of specialized pericytes, the podocytes, and an irreversible decrease in the density and function of intrarenal microvessels correlates with renal function decline in DN [2, 3]. Furthermore, podocyte injury is important in the development of DN in both type 1 and type 2 diabetes mellitus (DM) [4–6].

Heme oxygenase (HO) is the rate-limiting enzyme that degrades heme to produce carbon monoxide, iron, and biliverdin [7, 8]. HO is also anti-inflammatory, antioxidant, and antiapoptotic [9]. HO-1, the inducible form of HO, can be upregulated by many factors including oxidative stress [9]. As several reports have suggested that increased oxidative stress contributes to the development and progression of vascular complications in DM [10, 11], we focused on the putative role of HO-1 in the development of renal microvascular lesions during DN development.

## 2. Materials and Methods

### 2.1. Animals

*Hmox1^+/−^* deficient mice were kindly provided by Yet et al. [12]. Mice were on pure C57BL/6J genetic background. As *Hmox1* deficient (*Hmox1^−/−^*) mice show a high level of embryonic lethality [13], the effects of *Hmox1* haploinsufficiency were studied as means of assessing the intrinsic contribution of HO-1 in the development of renal microvascular lesions during diabetes mellitus. Deletion of one *Hmox1* allele (*Hmox1^+/−^*) reduced *Hmox1* mRNA expression by 70% in bone marrow and whole blood leukocytes.

### 2.2. Induction of Diabetes Mellitus with Streptozotocin (STZ)

*Hmox1^+/+^* (wild-type) or *Hmox1^+/−^* mice were made diabetic by streptozotocin (STZ) injection as previously described [14]. All mice were given free access to water and standard chow. Ten- to twelve-week-old males were rendered diabetic by STZ (Sigma-Aldrich, number S-0130) (100 mg/kg in sodium citrate buffer pH = 4.5) intraperitoneal injection on two consecutive days. Control mice received citrate buffer alone. Mice with fasting glycemia above 300 mg/dL were considered diabetic. Mice were killed 10 weeks after the induction of diabetes.

### 2.3. Assessment of Renal Function and Albuminuria

Urinary creatinine and blood urea nitrogen (BUN) concentrations were quantified spectrophotometrically by colorimetric methods. Urinary albumin excretion was measured with a specific ELISA assay (Cusabio, number CSB-E13878m).

### 2.4. Quantitative RT-PCR

Total RNA extraction of mice renal cortex was performed by using Qiazol (Qiagen), according to manufacturer's recommendations. RNA was reverse transcripted by using the Quantitect Reverse Transcription kit (Qiagen) according to the manufacturer's protocol. The Maxima SYBR Green/Rox qPCR mix (Thermo Scientific Fermentas) was used to amplify cDNA for 40 cycles on an ABI PRISM thermocycler. The comparative method of relative quantification (2-DDCT) was used to calculate the expression level of each target gene, normalized to *Ppia*. The oligonucleotide sequences are available upon request. The data are presented as the fold change in gene expression.

### 2.5. Histopathology and Immunohistochemistry

Kidneys were immersed in 10% formalin and embedded in paraffin. Sections (4 *μ*m thick) were stained with hematoxylin/eosin or Masson's trichrome and processed for histopathology or immunohistochemistry. For immunohistochemistry, paraffin-embedded sections were stained with rabbit anti-nytrotyrosine primary antibody (Upstate, number 06284, 1 : 200), rabbit anti-CD3 antibody (DAKO, number A0452), and rat anti-F4/80 antibody (AbD Serotec, number MCA497R) then the staining was revealed with Histofine reagent (Nichirei Biosciences) and slides were counterstained with hematoxylin. For immunofluorescence, paraffin-embedded sections were stained with a guinea pig anti-nephrin antibody (Progen) and a rat anti-CD31 antibody (Dianova, number SZ31) then the stainings were revealed with a goat anti-guinea pig alexa568-coupled antibody and a donkey anti-rat alexa488-coupled antibody (Invitrogen) and nuclei were counterstained with DAPI. Photomicrographs were taken with an Axiophot Zeiss photomicroscope. Staining surface quantifications were performed with a macro designed on ImageJ.

### 2.6. Electron Microscopy

Small pieces of renal cortex were fixed in Trump's fixative (EMS, number 11750) and embedded in Araldite M (Sigma-Aldrich, number 10951). Ultrathin sections were counterstained with uranyl acetate and lead citrate and examined with a transmission electron microscope.

### 2.7. Statistical Analyses

Data are expressed as mean ± SEM. Statistical analyses were calculated with GraphPad Prism (GraphPad software). Comparisons between 2 groups were performed with a 2-tailed Student *t*-test and between multiple groups were performed with one-way ANOVA followed by the Newman-Keuls test. A *p* value < 0.05 was considered statistically significant.

## 3. Results

**Figure 1:**
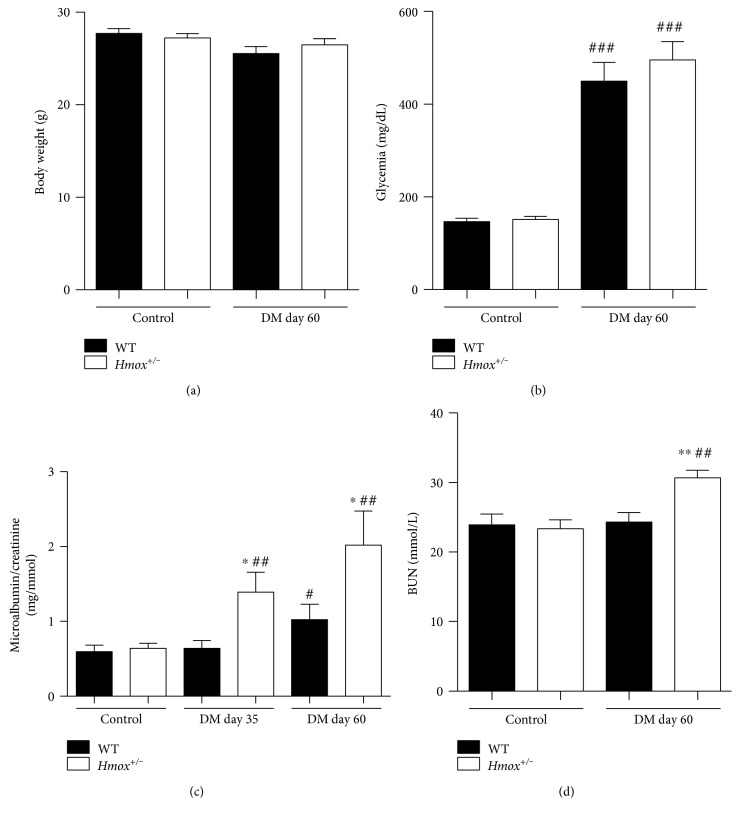
*Hmox1* deficiency sensitizes mice to diabetic nephropathy development. Body weight (a), fasted blood glycemia (b), microalbuminuria (c), and blood urea nitrogen (BUN) (d) in 20-week-old WT, *Hmox1*^+/−^, WT diabetic, and *Hmox1*^+/−^ diabetic mice. *n* = 10 to 12 mice. ∗ and ∗∗ indicate the statistical significance between *Hmox1*^+/−^ versus respective WT, that is, *Hmox*^+/−^ DM versus WT DM and *Hmox*^+/−^ control versus WT control. #, ##, and ### indicate the statistical significance between the same genotype versus respective control, that is, *Hmox*^+/−^ DM versus *Hmox*^+/−^ control, and WT DM versus WT control. ∗ and # indicate *p* < 0.05; ∗∗ and ## indicate *p* < 0.01.

10 weeks after STZ injection, both wild-type (WT) and *Hmox1^+/−^* mice had nonsignificant weight loss (Figure 1(a)) and fasting blood glucose > 400 mg/dL (Figure 1(b)). Both groups of diabetic mice developed features of mild nephropathy—microalbuminuria and elevated blood urea nitrogen (BUN) (Figures 1(c) and 1(d)). Importantly, both were significantly higher in *Hmox1^+/−^* diabetic mice than in controls (Figures 1(c) and 1(d)).

**Figure 2:**
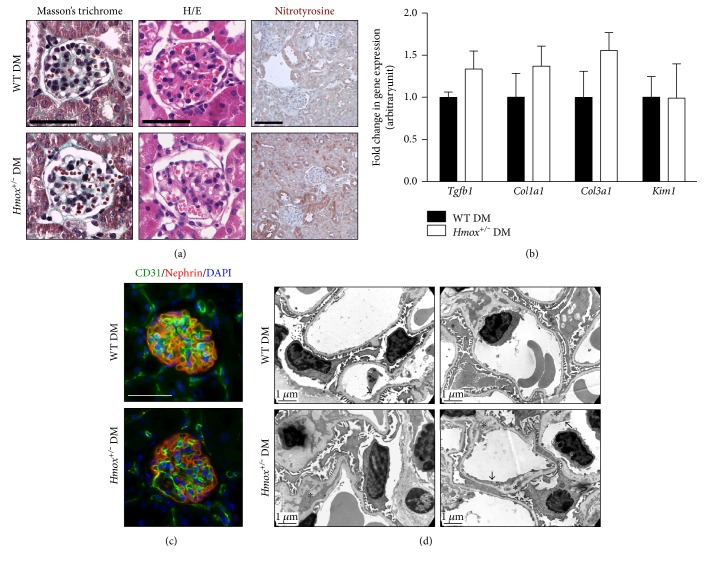
Mice with *Hmox* haploinsufficiency develop glomerular microvascular lesions under diabetes. (a) Representative images of Masson's trichrome staining (left panel), hematoxylin/eosin staining (middle panel), and nitrotyrosine immunohistochemistry (brown, right panel) in 20-week-old WT diabetic and *Hmox^+/−^* diabetic mice. Scale bar 50 *μ*m (left and middle panel) and 100 *μ*m (right panel). (b) qPCR analysis of *Tgfb1*, *Col1a1*, *Col3a1*, and *Kim1* mRNA expression in renal cortex extracts from 20-week-old WT diabetic and *Hmox^+/−^* diabetic mice. Data represent means ± sem of 5-6 mice. (c) Endothelial cells (CD31, green) and podocyte (nephrin, red) were stained in 20-week-old WT diabetic and *Hmox^+/−^* diabetic mice. Scale bar 50 *μ*m. Nuclei were stained with DAPI (blue). (d) Representative photomicrographs of transmission electron microscopy sections of glomerular capillary loops of 20-week-old WT diabetic and *Hmox*^+/−^ diabetic mice, showing loss of endothelial fenestrations (∗) and GBM inclusions (arrows) in *Hmox*^+/−^ diabetic mice. Scale bar 1 *μ*m.

In keeping with increased oxidative stress, renal staining for nitrotyrosine was greater in *Hmox1^+/−^* diabetic mice compared to WT (Figure 2(a)). Taken together, these results demonstrate that *Hmox1* haploinsufficiency sensitizes mice to DN development and that HO-1 exerts protective renal effects, partly through its antioxidant effects.

Control WT and *Hmox1*^+/−^ mice showed no functional and histological differences at baseline or after 60 days. DN is strongly linked to loss of integrity of the glomerular filtration barrier, particularly podocyte foot process effacement and alterations in glomerular endothelial cells. Histological analysis revealed similar levels of glomerulosclerosis in both diabetic WT and *Hmox1*^+/−^ groups (Figure 2(a)) (32.4 ± 9.2% versus 37.3 ± 7.6% of glomeruli with mesangial expansion) and no difference in tubular injury and tubulointerstitial fibrosis. These results were confirmed by qPCR analysis showing similar levels of expression of mRNAs implicated in fibrosis such as *Tgfb1*, *Col1a1*, and *Col3a1* and of the tubular injury marker *Kim1* (Figure 2(b)).

Interestingly, *Hmox1*^+/−^ diabetic mice had more glomerular microvascular lesions (capillary dilatations) compared to diabetic controls (41.7 ± 8.5% versus 18.3 ± 4.0% of glomeruli, respectively, *p* < 0.01) (Figure 2(a)). This was confirmed on CD31 stained kidneys (Figure 2(c)). In addition, electron microscopy revealed pronounced glomerular basement membrane (GBM) thickening and focal reduplication in *Hmox1*^+/−^ diabetic glomeruli compared to those from WT mice (Figure 2(d)). Endothelial damage was prominent with loss of endothelial fenestrations and cell thickening only (Figure 2(d)). Altered podocyte ultrastructural defects such as foot process broadening and effacement were more prevalent in glomeruli from *Hmox1*^+/−^ diabetic mice compared to diabetic controls (Figure 2(d)).

**Figure 3:**
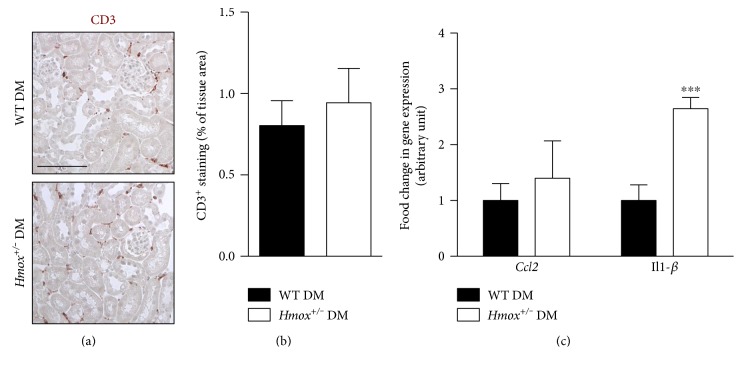
HO-1 does not modulate renal inflammatory cell infiltration in STZ-induced diabetes. (a) Representative images of CD3 immunohistochemistry (brown) in 20-week-old WT diabetic and *Hmox^+/−^* diabetic mice. Scale bar 50 *μ*m. (b) Percentage of tissue area stained for CD3. Data represent means ± sem of 5-6 mice. (c) qPCR analysis of *Ccl2* and *IL-1β* mRNA expression in renal cortex extracts from 20-week-old WT diabetic and *Hmox^+/−^* diabetic mice. Data represent means ± sem of 5-6 mice. ^∗∗∗^*p* < 0.001, *Hmox^+/−^* DM versus WT DM.

As HO-1 also exerts anti-inflammatory effects, we explored renal inflammation in our model. We did not detect F4/80^+^ macrophages in the diabetic kidneys (not shown) and only few CD3^+^ lymphocytes, with no difference between WT and *Hmox^+/−^* diabetic mice (Figure 3), thus suggesting that HO-1 does not modulate inflammatory cells in that model. Correlating with such results, we observed similar mRNA expression of *Ccl2*, coding for the chemokine MCP-1 (Figure 3). Other mRNAs like *Trp53*, coding for TNF*α*, *Il10*, and *Il6*, were undetectable in diabetic kidneys of both WT and *Hmox^+/−^* mice (not shown). Interestingly, *IL-1β* mRNA expression was increased in *Hmox^+/−^* diabetic mice when compared to WT diabetic mice (Figure 3).

## 4. Discussion

As only indirect evidence suggests that HO-1 activity may limit progression of DN, we sought to investigate the specific role of this enzyme in experimental DN using a model of genetic *Hmox1* deficiency. Various levels of HO-1 expression and activity have been reported in tissues from diabetic animals [15] with no definitive conclusion.

Administration of HO-1 inducers (hemin, zinc protoporphyrin, cobalt protoporphyrin, and tert-butylhydroquinone) protects from renal damage, particularly glomerular lesions during DN development [16–19]. Meanwhile, the specificity of these compounds has been challenged [20, 21]. Thus, direct and specific alteration of HO-1 such as the one achieved with ablation of the *Hmox1* gene is needed. The importance of HO-1 in vascular protection is demonstrated by the spontaneous severe endothelial damage observed in human cases of HO-1 deficiency and in *Hmox1^−/−^* mice [22, 23]. These studies suggest that HO-1 might exert its protective effects through its antioxidant, antiapoptotic, and anti-inflammatory properties.

Recently, Zheng and colleagues demonstrated that NRF2 stimulation limits the progression of DN by repressing ECM production and P21 expression [24]. Here, we found that HO-1 does not modulate tubulointerstitial fibrosis nor glomerulosclerosis during diabetes. As NRF2 induces HO-1 but also other proteins such as NQO1 and GST-1P, the benefit induced by NRF2 stimulation on ECM production could be independent on HO-1. These observations suggest that NRF2 and HO-1 could exert renoprotection through different targets and/or pathways and that stimulating both NRF2 and HO-1 during diabetes could provide complementary beneficial effects on both kidney tubulointerstitial compartments and glomerular microvasculature.

We have shown that HO-1 protects the glomerular microcirculation during diabetes. HO-1 haploinsufficiency sensitized mice to the development of glomerular injury—especially the glomerular endothelial cells—during DN. Although moderate improvement in insulin sensitivity has been shown in diabetic mice following HO-1 induction [25–27], we found no difference in fasting glucose levels in HO-1 deficient or control animals supporting direct vascular actions of HO-1. Interestingly, *IL-1β* mRNA expression was increased in *Hmox^+/−^* diabetic mice. (i) Few ancient studies showed that the main source of IL-1*β* in renal pathology is the glomerulus itself (probably podocytes) [28, 29]. (ii) IL-1*β*, but also IL-18, a cytokine secreted by the inflammasome concomitantly to IL-1*β*, exerts several deleterious effects on endothelial cells such as increased vascular permeability for IL-1*β* [30–32] and induction of apoptosis and of adhesion molecule for IL-18 [33, 34]. While the deleterious role of IL-1*β* in renal cells during diabetes is not clearly established, IL-18 deleterious role is clearly demonstrated [35]. Thus, diabetic glomerular microvascular injuries induced by HO-1 partial deficiency might be due, at least partly, to increased inflammasome activation. Supporting this hypothesis, a recent study showed that hemin inhibits NLRP3 inflammasome activation in a model of lung injury, through HO-1 activation [36]. Investigating the mechanisms for this should be the area of future research.

Peroxynitrite is a potent oxidant produced by rapid interaction between superoxide anion and nitric oxide radicals and induces oxidative stress and cell death. Beckman and colleagues proposed that the production of peroxynitrite may play a role in endothelial damage *in vivo* [37]. Since then, robust evidence supports the possibility of the *in vivo* formation of this molecule [38]. Peroxynitrite contributes to endothelial damage through a mechanism involving ER stress [39].

We found markedly less nitrotyrosine staining in kidneys from wild-type diabetic mice than in *Hmox1^+/−^* diabetic animals, suggesting that either superoxide anion production and/or nitric oxide radical formation was blunted by HO-1 activity. Nitrosylation of tyrosine moieties of cellular proteins in the kidneys of patients with DM has been demonstrated. Moreover, the absence of nitrotyrosine in other types of nephropathies indicates that this pathway is specific for DN [40]. HO-1 activity may both prevent peroxynitrite formations and may also display cytoprotective action through generation of carbon monoxide that protects cells from peroxynitrite-induced apoptotic death [41]. Interestingly, HO-1 induction by peroxynitrite is also a defense mechanism [42]. Although one cannot rule out potential nonenzymatic effects of HO-1 [43], a potent antioxidant role is likely.

HO-1 produces biliverdin/bilirubin and CO. As all these products have been found to exert anti-inflammatory, antiapoptotic, and antioxidant effects, we cannot, in the present study, determine if the observed changes in our *Hmox^+/−^* diabetic mice are specifically due to one of this product, if it is a combination of all of them, or if it is an independent protective effect of HO-1.

## 5. Conclusion

In summary, using for the first time a specific *Hmox1* gene-targeting approach, we highlight a role of HO-1 in protecting glomerular endothelial cells and podocytes against progressive DN. We show that mice with a heterozygous deletion of *Hmox1* alleles are prone to accelerated diabetes-induced structural and ultrastructural damage of the glomerular capillary with accentuated microalbuminuria. Interestingly, we also found the first evidence that HO-1 drives protection from nitrative stress. Our results support the hypothesis that HO-1 is an important modulator of microvascular endothelial injury during DN and thus an interesting target for DN therapy.

## Abbreviations

HO-1: Heme oxygenase-1 protein
*Hmox1*: Heme oxygenase gene
*Hmox1^−/−^*: *Hmox1* deficient (gene knock-out)
*Hmox1^+/−^*: *Hmox1* haploinsufficiency (with only a single functional copy of the *Hmox1* gene, with the other copy inactivated by mutation)
STZ: Streptozotocin
BUN: Blood urea nitrogen
DM: Diabetes mellitus
DN: Diabetic nephropathy
WT: Wild-type
ER stress: Endoplasmic reticulum (ER) stress.

## References

[1] Van Buren P. N., Toto R. (2013). Current update in the management of diabetic nephropathy. *Current Diabetes Reviews*.

[2] Ishimura E., Nishizawa Y., Kawagishi T. (1997). Intrarenal hemodynamic abnormalities in diabetic nephropathy measured by duplex Doppler sonography. *Kidney International*.

[3] Lindenmeyer M. T., Kretzler M., Boucherot A. (2007). Interstitial vascular rarefaction and reduced VEGF-A expression in human diabetic nephropathy. *Journal of the American Society of Nephrology*.

[4] Najafian B., Alpers C. E., Fogo A. B. (2011). Pathology of human diabetic nephropathy. *Contributions to Nephrology*.

[5] Stitt-Cavanagh E., MacLeod L., Kennedy C. (2009). The podocyte in diabetic kidney disease. *The Scientific World Journal*.

[6] Reddy G. R., Kotlyarevska K., Ransom R. F., Menon R. K. (2008). The podocyte and diabetes mellitus: is the podocyte the key to the origins of diabetic nephropathy?. *Current Opinion in Nephrology and Hypertension*.

[7] Tenhunen R., Marver H. S., Schmid R. (1968). The enzymatic conversion of heme to bilirubin by microsomal heme oxygenase. *Proceedings of the National Academy of Sciences of the United States of America*.

[8] Maines M. D. (1988). Heme oxygenase: function, multiplicity, regulatory mechanisms, and clinical applications. *The FASEB Journal*.

[9] Abraham N. G., Kappas A. (2005). Heme oxygenase and the cardiovascular-renal system. *Free Radical Biology & Medicine*.

[10] Dandona P., Thusu K., Cook S. (1996). Oxidative damage to DNA in diabetes mellitus. *The Lancet*.

[11] Forbes J. M., Coughlan M. T., Cooper M. E. (2008). Oxidative stress as a major culprit in kidney disease in diabetes. *Diabetes*.

[12] Yet S. F., Perrella M. A., Layne M. D. (1999). Hypoxia induces severe right ventricular dilatation and infarction in heme oxygenase-1 null mice. *The Journal of Clinical Investigation*.

[13] Zhao H., Wong R. J., Kalish F. S., Nayak N. R., Stevenson D. K. (2009). Effect of heme oxygenase-1 deficiency on placental development. *Placenta*.

[14] Lenoir O., Jasiek M., Hénique C. (2015). Endothelial cell and podocyte autophagy synergistically protect from diabetes-induced glomerulosclerosis. *Autophagy*.

[15] Grochot-Przeczek A., Dulak J., Jozkowicz A. (2010). Heme oxygenase-1 in neovascularisation: a diabetic perspective. *Thrombosis and Haemostasis*.

[16] Ptilovanciv E. O., Fernandes G. S., Teixeira L. C. (2013). Heme oxygenase 1 improves glucoses metabolism and kidney histological alterations in diabetic rats. *Diabetology & Metabolic Syndrome*.

[17] Lee S. C., Han S. H., Li J. J. (2009). Induction of heme oxygenase-1 protects against podocyte apoptosis under diabetic conditions. *Kidney International*.

[18] Li H., Zhang L., Wang F. (2011). Attenuation of glomerular injury in diabetic mice with tert-butylhydroquinone through nuclear factor erythroid 2-related factor 2-dependent antioxidant gene activation. *American Journal of Nephrology*.

[19] Elmarakby A. A., Faulkner J., Baban B., Saleh M. A., Sullivan J. C. (2012). Induction of hemeoxygenase-1 reduces glomerular injury and apoptosis in diabetic spontaneously hypertensive rats. *American Journal of Physiology - Renal Physiology*.

[20] Loboda A., Jazwa A., Wegiel B., Jozkowicz A., Dulak J. (2005). Heme oxygenase-1-dependent and -independent regulation of angiogenic genes expression: effect of cobalt protoporphyrin and cobalt chloride on VEGF and IL-8 synthesis in human microvascular endothelial cells. *Cellular and Molecular Biology*.

[21] Wang S., Avery J. E., Hannafon B. N., Lind S. E., Ding W. Q. (2013). Zinc protoporphyrin suppresses cancer cell viability through a heme oxygenase-1-independent mechanism: the involvement of the Wnt/β-catenin signaling pathway. *Biochemical Pharmacology*.

[22] Yachie A., Niida Y., Wada T. (1999). Oxidative stress causes enhanced endothelial cell injury in human heme oxygenase-1 deficiency. *Journal of Clinical Investigation*.

[23] Poss K. D., Tonegawa S. (1997). Heme oxygenase 1 is required for mammalian iron reutilization. *Proceedings of the National Academy of Sciences of the United States of America*.

[24] Zheng H., Whitman S. A., Wu W. (2011). Therapeutic potential of Nrf2 activators in streptozotocin-induced diabetic nephropathy. *Diabetes*.

[25] Liu J., Wang L., Tian X. Y. (2015). Unconjugated bilirubin mediates heme oxygenase-1-induced vascular benefits in diabetic mice. *Diabetes*.

[26] Nicolai A., Li M., Kim D. H. (2009). Heme oxygenase-1 induction remodels adipose tissue and improves insulin sensitivity in obesity-induced diabetic rats. *Hypertension*.

[27] Ndisang J. F., Jadhav A. (2009). Up-regulating the hemeoxygenase system enhances insulin sensitivity and improves glucose metabolism in insulin-resistant diabetes in Goto-Kakizaki rats. *Endocrinology*.

[28] Tesch G. H., Yang N., Yu H. (1997). Intrinsic renal cells are the major source of interleukin-1 beta synthesis in normal and diseased rat kidney. *Nephrology Dialysis Transplantation*.

[29] Niemir Z. I., Stein H., Dworacki G. (1997). Podocytes are the major source of IL-1*α* and IL-1*β* in human glomerulonephritides. *Kidney International*.

[30] Campbell W. N., Ding X., Goldblum S. E. (1992). Interleukin-1 alpha and -beta augment pulmonary artery transendothelial albumin flux in vitro. *American Journal of Physiology - Lung Cellular and Molecular Physiology*.

[31] Du L., Dong F., Guo L. (2015). Interleukin-1β increases permeability and upregulates the expression of vascular endothelial-cadherin in human renal glomerular endothelial cells. *Molecular Medicine Reports*.

[32] Zhu W., London N. R., Gibson C. C. (2012). Interleukin receptor activates a MYD88–ARNO–ARF6 cascade to disrupt vascular stability. *Nature*.

[33] Morel J. C., Park C. C., Zhu K., Kumar P., Ruth J. H., Koch A. E. (2002). Signal transduction pathways involved in rheumatoid arthritis synovial fibroblast interleukin-18-induced vascular cell adhesion molecule-1 expression. *Journal of Biological Chemistry*.

[34] Zhou G., Zhou Z., Ge S. (2009). IL-18 accelerates the cell apoptosis by up-regulating cysteinyl leukotriene 2 receptor expression in human umbilical vein endothelial cells at the early stage of administration. *Vascular Pharmacology*.

[35] Elsherbiny N. M., Al-Gayyar M. M. (2016). The role of IL-18 in type 1 diabetic nephropathy: the problem and future treatment. *Cytokine*.

[36] Luo Y. P., Jiang L., Kang K. (2014). Hemin inhibits NLRP3 inflammasome activation in sepsis-induced acute lung injury, involving heme oxygenase-1. *International Immunopharmacology*.

[37] Beckman J. S., Beckman T. W., Chen J., Marshall P. A., Freeman B. A. (1990). Apparent hydroxyl radical production by peroxynitrite: implications for endothelial injury from nitric oxide and superoxide. *Proceedings of the National Academy of Sciences of the United States of America*.

[38] Szabo C. (2009). Role of nitrosative stress in the pathogenesis of diabetic vascular dysfunction. *British Journal of Pharmacology*.

[39] Dickhout J. G., Hossain G. S., Pozza L. M., Zhou J., Lhotak S., Austin R. C. (2005). Peroxynitrite causes endoplasmic reticulum stress and apoptosis in human vascular endothelium: implications in atherogenesis. *Arteriosclerosis, Thrombosis, and Vascular Biology*.

[40] Thuraisingham R. C., Nott C. A., Dodd S. M., Yaqoob M. M. (2000). Increased nitrotyrosine staining in kidneys from patients with diabetic nephropathy. *Kidney International*.

[41] Li M. H., Cha Y. N., Surh Y. J. (2006). Carbon monoxide protects PC12 cells from peroxynitrite-induced apoptotic death by preventing the depolarization of mitochondrial transmembrane potential. *Biochemical and Biophysical Research Communications*.

[42] Li M. H., Cha Y. N., Surh Y. J. (2006). Peroxynitrite induces HO-1 expression via PI3K/Akt-dependent activation of NF-E2-related factor 2 in PC12 cells. *Free Radical Biology & Medicine*.

[43] Dulak J., Jozkowicz A. (2014). Novel faces of heme oxygenase-1: mechanisms and therapeutic potentials. *Antioxidants & Redox Signaling*.

